# cGAS-STING pathway as a promising target for digestive diseases: insights from natural plant products

**DOI:** 10.3389/fnut.2025.1594120

**Published:** 2025-06-20

**Authors:** Dan Long, Chenhan Mao, Ying Zhu, Yin Xu

**Affiliations:** ^1^The First Hospital of Hunan University of Chinese Medicine, Changsha, Hunan, China; ^2^Affiliated Hospital of Integrated Traditional Chinese and Western Medicine, Nanjing University of Chinese Medicine, Nanjing, Jiangsu, China

**Keywords:** cGAS, STING, digestive diseases, traditional Chinese medicine, active ingredients

## Abstract

Digestive diseases remain a major challenge to public health systems globally. Cyclic GMP-AMP synthase (cGAS) and stimulator of interferon gene (STING) play important roles in innate immunity as well as inflammatory responses. Dysregulation of the cGAS-STING pathway has been demonstrated to be an important pathogenetic mechanism in diverse gastrointestinal diseases. Therefore, targeting the cGAS-STING pathway is a potential therapeutic strategy for digestive diseases. Encouragingly, increasing studies have revealed that natural plant products are promising candidates for the treatment of digestive disorders. This review discussed the research progress of cGAS-STING pathway mediating common digestive diseases, including inflammatory bowel disease, liver disease, colorectal cancer, gastric cancer, esophageal cancer, pancreatitis, and pancreatic cancer. In addition, we systematically summarized recent advances in the treatment of gastrointestinal disorders with phytochemicals that target the cGAS-STING pathway.

## 1 Introduction

The digestive system is the system with the largest number of organs in the human body. Diseases of the digestive system are common and frequent diseases that seriously jeopardize physical and mental health, and severely affect the quality of life in patients. Digestive diseases mainly include gastrointestinal diseases, liver and gallbladder diseases, pancreatic diseases, and so on. The organs of the digestive system can influence metabolic, immune, and endocrine functions, interacting with other systems of the body (1). As a result, digestive disorders are complex and difficult to treat. Unfortunately, digestive disorders have become a major cause of the current burden of disease globally and are posing a serious threat to human health (2). Therefore, it is of great urgency to clarify their pathological mechanisms pathogenesis to identify more effective therapeutic targets and strategies.

In 2012, Wu et al. (3) demonstrated that cyclic GMP-AMP (cGAMP) functions as an endogenous second messenger that binds and activates the stimulator of interferon genes (STING), linking DNA sensing to type I interferon responses. Further, Sun et al. (4) identified cyclic GMP-AMP synthase (cGAS) as the key enzyme responsible for DNA sensing and cGAMP production. cGAS and the endoplasmic reticulum (ER)-associated protein STING are essential for initiating the innate immune response to cytosolic DNA (5, 6). Importantly, the cGAS-STING pathway acts as a double-edged sword. It is essential for host defense against infections and tumor surveillance by inducing type I interferons (IFN-I) and antiviral responses (7). However, aberrant activation of the cGAS-STING pathway by self DNA can trigger chronic inflammation, autoimmunity, and various diseases (8, 9). Recently, the cGAS-STING pathway has emerged as a major focus of research in the areas of anti-tumor immunity, autoimmune diseases, and inflammatory diseases (10–12). Increasing evidence indicates that dysfunctions within the cGAS-STING signaling potentially mediate the pathogenesis of diverse digestive diseases, such as inflammatory bowel disease (IBD), non-alcoholic fatty liver disease (NAFLD), and colorectal cancer (CRC) (13–16). Consequently, targeting cGAS-STING signaling may represent a therapeutic strategy for digestive disorders. Traditional Chinese Medicine (TCM) has been practiced in China for thousands of years in the prevention, treatment, and diagnosis of diseases. Encouragingly, numerous studies have demonstrated the potential of herbal active components to cure digestive problems by targeting the cGAS-STING pathway (17–19). Nevertheless, few studies have systematically summarized the modulatory effects of herbal medicines and their main components on the cGAS-STING pathway. In this review, the role of cGAS-STING pathway in digestive diseases was discussed. In addition, we thoroughly reviewed the potential of natural plant compounds that target the cGAS-STING pathway for the treatment of gastrointestinal diseases. This review aims to offer diverse perspectives on the development of therapeutic medicines for digestive disorders.

## 2 The cGAS-STING signaling pathway

**Figure 1:**
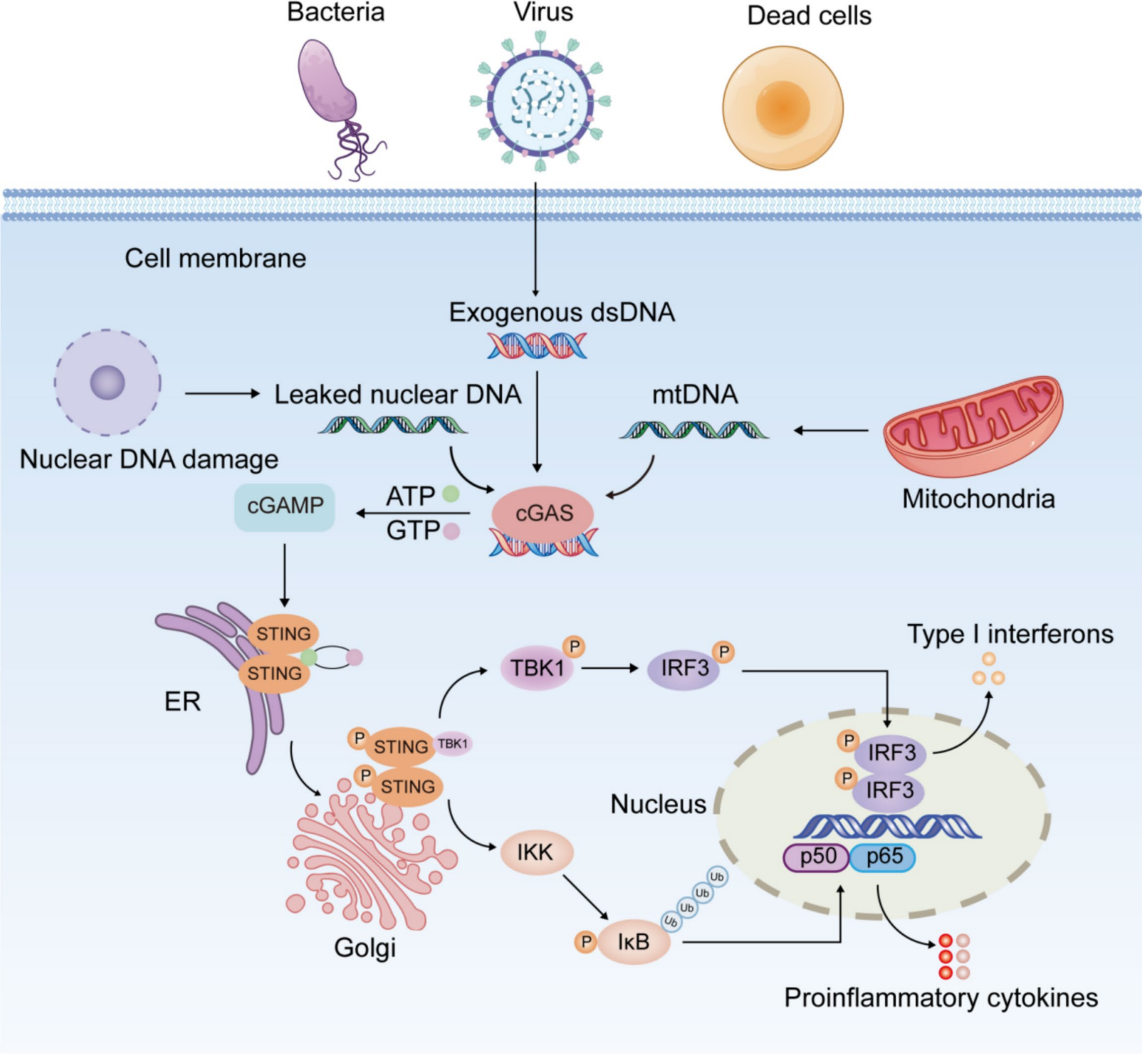
Schematic diagram of the cGAS-STING signaling pathway.

The most striking feature of the cGAS-STING signaling pathway is that its activator is DNA, the most essential element of life, rather than the causative agent of a specific pathogen. cGAS is an innate immune sensor which recognizes various cytoplasmic double-stranded DNA (dsDNA) (20). cGAS consists of two binding structural domains and a nucleotidyltransferase structural domain. It has been found that the binding of cGAS to DNA is not related to DNA-specific base sequences, but rather to the sugar backbone of dsDNA (4). In In healthy individuals, DNA is replicated and transcribed into mRNA, which is then translated into proteins. This process is tightly regulated to ensure controlled cell growth and genomic stability. However, when the extent of DNA damage exceeds the repair capacity of the body’s repair system, DNA entering the cytoplasm activates the cGAS-STING signaling pathway (Figure 1). Damaged self-DNA, microbial DNA, and necrotic cell debris represent crucial elements that trigger the activation of cGAS-STING pathway. Under physiological conditions, cGAS specifically detects exogenous pathogenic DNA (such as viral or bacterial DNA), initiating antimicrobial immune responses for pathogen clearance. In contrast, abnormal accumulation of endogenous DNA may induce aberrant activation of cGAS-STING signaling, potentially leading to inflammatory pathology. Both nuclear and mitochondrial DNA (mtDNA) are vulnerable to damage under cellular stress. When released into the cytoplasm due to membrane rupture or defective clearance, these DNA species become potent activators of the cGAS-STING pathway.

The cGAS-STING pathway exhibits a dual role in inflammation. Its physiological activation is critical for innate immunity by clearing pathogens through IFN-mediated responses, while excessive or prolonged signaling promotes inflammation, tissue damage, and autoimmune disorders (21, 22). When aberrant DNA accumulated in the cytoplasm is recognized by cGAS, cGAS binds to it and induces a conformational change in the active site of cGAS. Activated cGAS catalyzes the synthesis of 2′,3′-cyclic guanosine monophosphate-adenosine monophosphate (2′,3′-cGAMP) from adenosine triphosphate (ATP) and guanosine triphosphate (GTP) (23). As a second messenger, cGAMP binds to STING anchored in the ER membrane, triggering a conformational transition and dimerization of STING and prompting the migration of STING from the ER to the Golgi (24). The STING dimer recruits and phosphorylates TANK-binding kinase 1 (TBK1), followed by the formation of the STING-TBK1 complex. Phosphorylated TBK1 in turn phosphorylates and activates interferon regulatory factor 3 (IRF3) (24). P-IRF3 further dimerizes and translocates into the nucleus, activating the transcription of IFN-I (interferon α/β) and interferon-stimulated genes (25). Furthermore, STING activates classical and non-classical nuclear factor kappa-B (NF-κB) pathway. The canonical NF-κB pathway is triggered by various stimuli, including diverse cytokine receptors, pattern recognition receptors, tumor necrosis factor (TNF) receptors, T-cell receptors, and B-cell receptors (26). The primary mechanism for canonical NF-κB activation is the inducible degradation of inhibitor of NF-κB (IκBα), triggered by its site-specific phosphorylation by IκB kinase (IKK) complex (27). IκBα degradation allows rapid nuclear translocation of p50/p65 and p50/c-Rel, the major canonical NF-κB dimers. Unlike the canonical NF-κB pathway, the non-canonical pathway responds selectively to stimulation through specific TNFR superfamily members, notably LTβR, BAFFR, CD40, and RANK (28). Non-canonical NF-κB activation depends on NF-κB-inducing kinase (NIK)-mediated phosphorylation of IKKα dimers. IKKα activation induces p100 processing into p52, facilitating the nuclear translocation of p52/RelB heterodimers (29). Activation of the canonical pathway is primarily linked to inflammation, whereas the non-canonical pathway is critical for cell development and organogenesis. TBK1 serves as a critical kinase in STING-induced NF-κB activation by directly modulating IKK complex activity (30). STING-NF-κB response may be mediated by TBK1 or IKKε (31). After activation of the NF-κB pathway, the heterodimer P65/P50 translocates into the nucleus and induces the expression of downstream inflammatory mediators like interleukin-1β (IL-1β), interleukin-6 (IL-6), and TNF-α (32, 33). In dendritic cells, non-canonical NF-κB signaling is triggered by the STING-mediated DNA-sensing pathway (34).

The cGAS-STING signaling pathway exhibits extensive expression across immune cells, non-immune cells, and cancer cells. Interestingly, numerous recent researches have further observed that the cGAS-STING signaling is involved in the modulation of diverse cell death mechanisms, including pyroptosis, apoptosis, necroptosis, autophagy, and ferroptosis (35–39). For example, STING signaling induces apoptosis and pyroptosis in cardiomyocytes through NOD-like receptor protein 3 (NLRP3) inflammasome activation (35). Besides, STING activates NLRP3-mediated pyroptosis in intestinal epithelial cells (IEC) (38). Interestingly, cGAS binds directly to Beclin-1 in IEC, thereby enhancing autophagy and suppressing apoptosis (36). A recent study found that neutrophil extracellular traps (NETs), taken up by alveolar epithelial cells (AEC) via endocytosis, activate the cGAS-STING pathway, ultimately leading to AEC necroptosis in mice (37). Additionally, activation of the STING signaling pathway induces lipid peroxide accumulation, promoting ferroptosis in renal tubular epithelial cells and aggravating renal injury (39).

## 3 The role of cGAS-STING pathway in digestive disorders

### 3.1 cGAS-STING pathway and IBD

**Figure 2:**
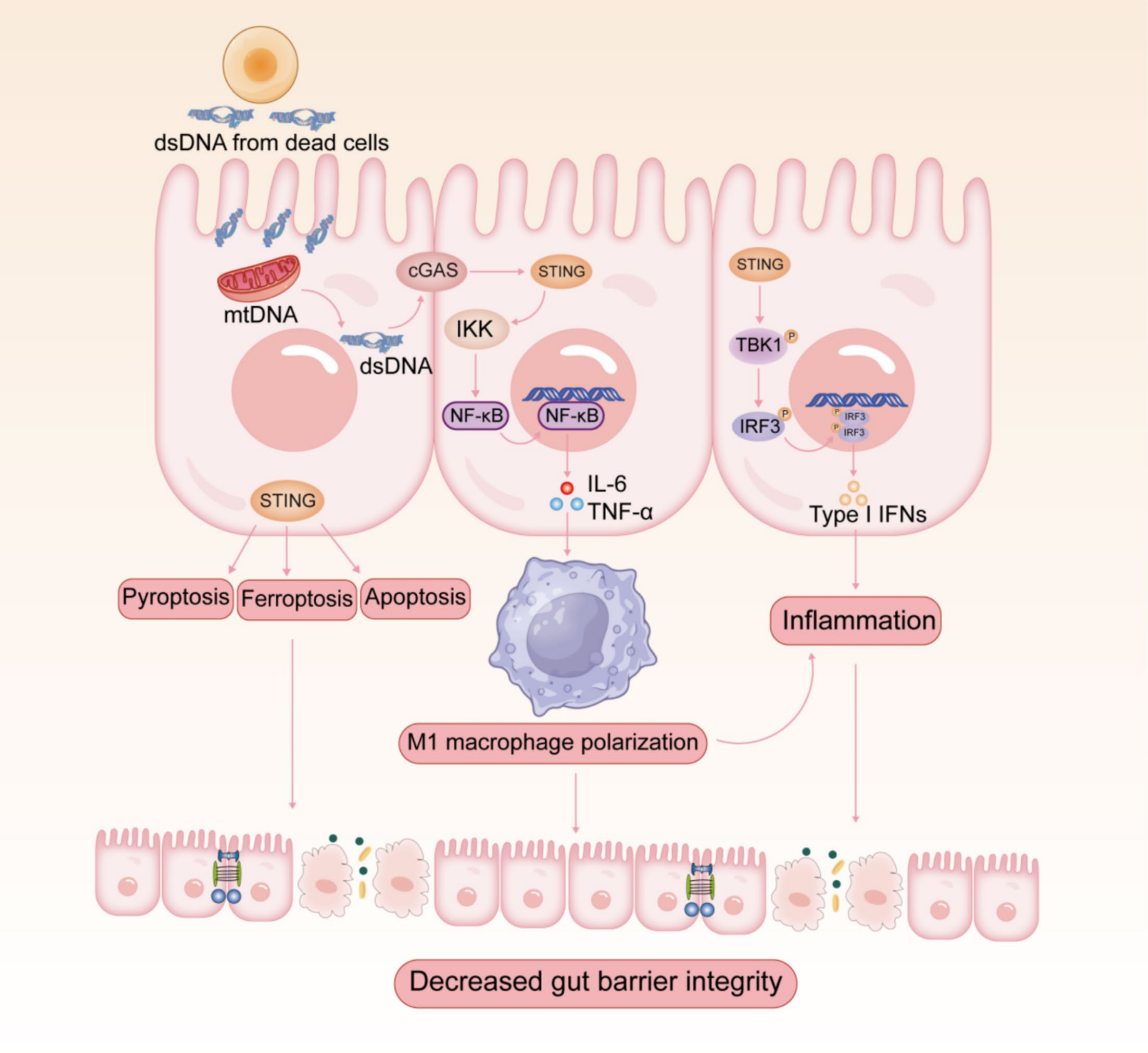
Potential role of cGAS-STING signaling pathway in the pathogenesis of IBD.

Increasing evidence revealed that the overactivation of cGAS-STING pathway triggers abnormal innate immune responses, which may be associated with the development and progression of multiple autoimmune diseases (10, 11). IBD is one of the most common autoimmune diseases, which mainly include ulcerative colitis (UC) and Crohn’s disease (CD). Clinical evidence suggests that the expression levels of cGAS and STING are increased in the colon of patients with UC and CD (15, 36, 40, 41). Since intestinal mucosal injury possibly involves cell death and DNA release, cGAS-STING, the DNA-sensing pathway, is relevant to the pathogenesis of IBD (Figure 2). IBD patients and mice show elevated levels of extracellular DNA in serum and tissues (42–44), which is mainly derived from apoptotic body, necrotic cells, and NETs (44). cGAS-deficient mice were insensitive to dextran sulfate sodium (DSS)-induced colitis and suffered from lower disease severity (45). Furthermore, DSS-induced colitis was significantly mitigated by intraperitoneal injection of RU.521, a selective cGAS inhibitor, which improved weight loss, disease activity index score, shortened colon length, and histopathological manifestations (46). In contrast, the STING agonist 5,6-dimethylxanthenone-4-acetic acid (DMXAA) exacerbated DSS-induced colitis (47). As predicted, STING-deficient mice showed significantly milder symptoms in colitis (15, 16). Several reports have indicated that the stimulation of STING aggravated experimental colitis and facilitated the necroptosis of IECs, emphasizing the pathological effect of STING during the inflammatory process (47, 48). Additionally, the expression of STING and IFN-I signaling pathway is dramatically elevated in colonic tissues of patients with active UC (41). The constitutive activation of STING promotes spontaneous enteritis, progressive chronic intestinal inflammation, and intestinal fibrosis (49). The aberrant activation of cGAS-STING induces intestinal inflammation through activating downstream STING-IRF3 or NF-κB signaling pathways (50). In recent years, researchers have focused on the multiple roles of cGAS-STING signaling in IBD apart from interferons and cytokines, including autophagy, pyroptosis, necroptosis, and ferroptosis (38, 51). Interestingly, research has revealed that aberrant activation of the cGAS-STING pathway exacerbates UC by inducing pro-inflammatory macrophage phenotypic polarization (15). The pharmacological degradation of STING attenuates colitis by inhibiting M1-type macrophage polarization (38).

**Figure 3:**
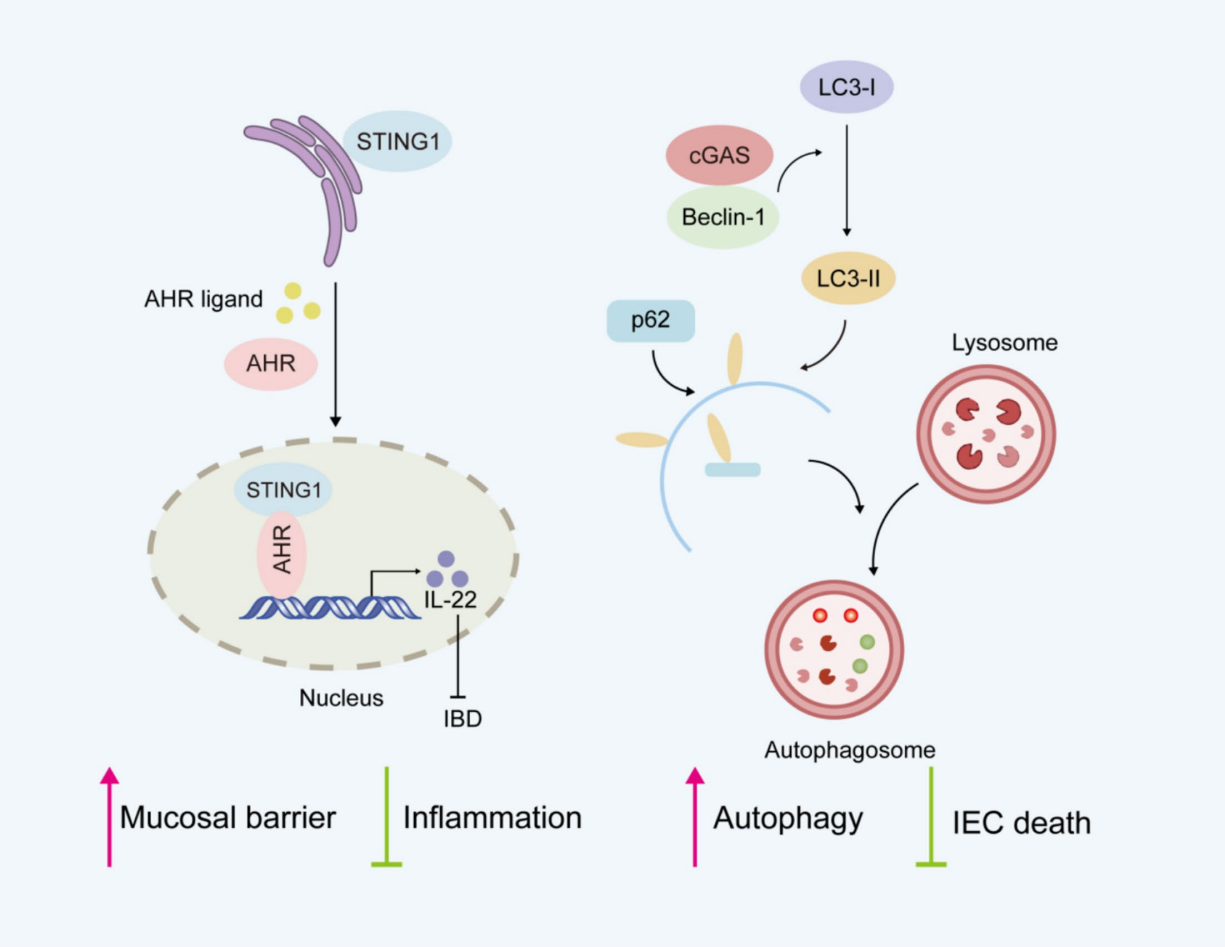
Potential protective role of cGAS-STING signaling pathway in intestinal homeostasis.

Currently, the role of cGAS-STING pathway in intestinal inflammation remains controversial. Seemingly paradoxically, complete STING deficiency (52) or cell-specific STING deletion (53) in splenic CD4 T cells increases the severity of colitis in DSS-induced colitis models. STING maintains intestinal homeostasis by regulating both the mucosal barrier and inflammation (Figure 3). STING-deficient mice show impaired mucosal barrier protection, with fewer goblet cells and reduced secretory IgA production (52). IgA plays a crucial role in maintaining intestinal homeostasis by limiting the translocation of commensal bacteria across the epithelial barrier (54). STING deficiency is also characterized by gut dysbiosis and a reduced proportion of intestinal regulatory T cells (52). Research indicates that STING activation in Th1 cells antagonizes Th cell-mediated pathology and attenuates colonic inflammation, supporting its protective function in intestinal homeostasis and colitis (53). The aryl hydrocarbon receptor (AhR), a ligand-activated transcription factor, plays an essential role in maintaining intestinal homeostasis (55). Considerable evidence indicates that AHR activation alleviates intestinal inflammation and enhances mucosal barrier repair, thereby mitigating IBD progression (56). Interestingly, the newly identified STING1-AHR nuclear axis represents a critical immunomodulatory mechanism where STING1 competitively coordinates both cytoplasmic DNA sensing and nuclear transcriptional programs (16). In contrast to its cytoplasmic functions, STING1 localizes to the nucleus and activates the transcription factor AHR. The STING1-AHR axis promotes IL-22 secretion from type 3 innate lymphoid cells (ILC3) and Th17 cells while inhibiting proinflammatory mediators, thereby maintaining mucosal immune homeostasis. Nuclear STING1 exhibits IFN-independent activity, which plays a critical role in attenuating intestinal inflammation and sustaining gut microbiota balance. Besides, cGAS was shown to prevent the death of IECs by upregulating Beclin-1-mediated autophagy (36). cGAS deficiency reduced the expression level of autophagic proteins, which led to worsened colitis.

Atrial natriuretic peptide was found to inhibit the STING pathway and repair intestinal barrier damage in the DSS-induced colitis model (57). Brefeldin A, a fungal metabolite identified as an inhibitor of protein trafficking (58), may ameliorate colitis by suppressing the activation of cGAS-STING pathway and NLRP inflammasome (45). Furthermore, low-dose ganciclovir, an antiviral nucleoside analog, ameliorates DSS-induced UC in mice by suppressing STING signaling in colonic macrophages (15). Overall, STING may have a role in maintaining intestinal homeostasis under physiological conditions, but it is more emphasized that aberrant activation of the cGAS-STING pathway plays a proinflammatory role in the context of intestinal inflammation (59). While the cGAS-STING pathway has been implicated in IBD pathogenesis, its precise mechanisms and therapeutic potential as a key innate immune signaling in intestinal inflammation require further elucidation.

### 3.2 cGAS/STING pathway and liver diseases

**Figure 4:**
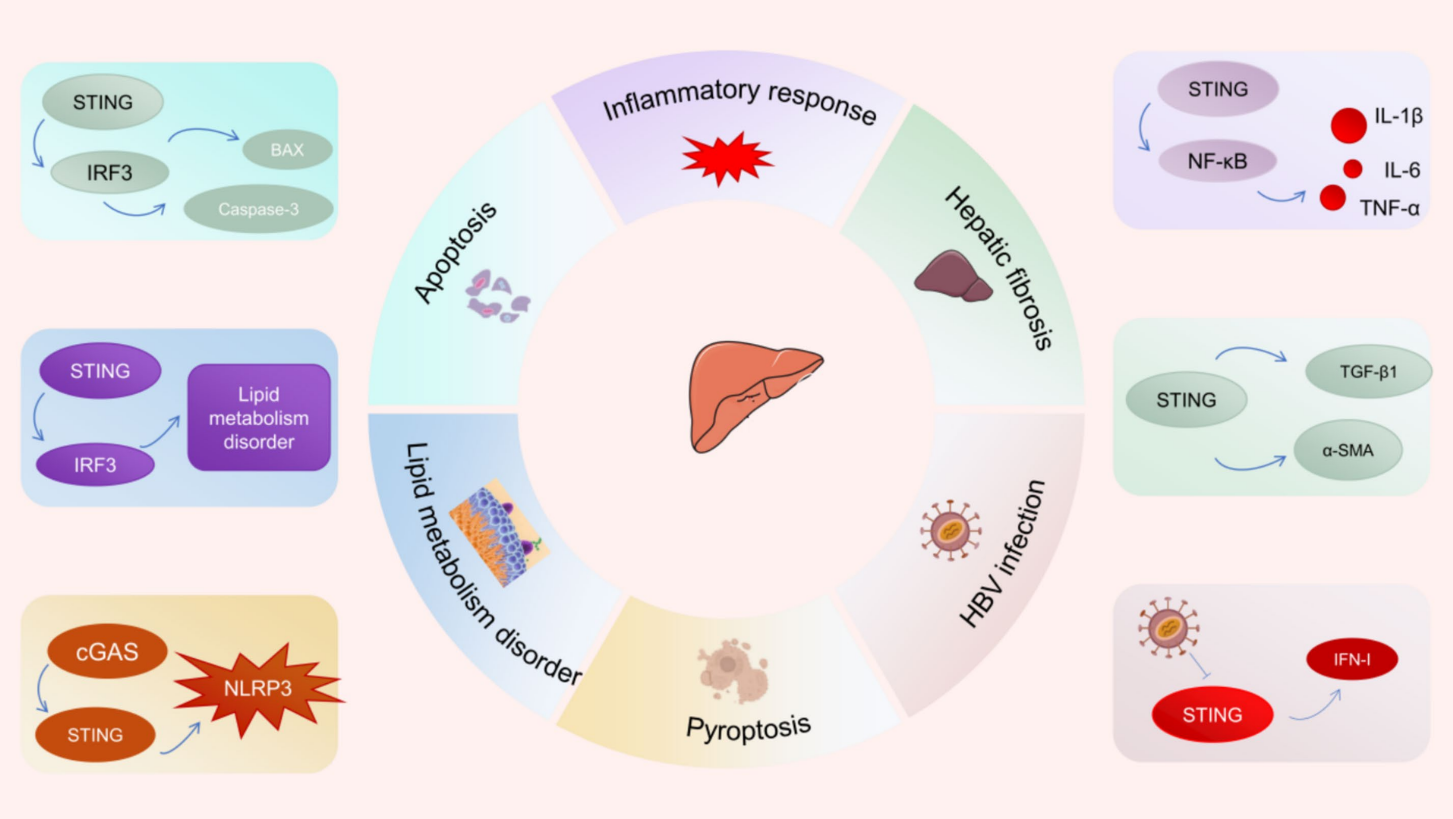
Potential role of cGAS-STING signaling pathway in liver diseases.

More and more studies have revealed that the cGAS-STING pathway is involved in the pathogenesis and progression of several liver diseases (Figure 4) (60–62). NAFLD is the most common chronic liver disease around the world. Recent studies have revealed that STING signaling is over-activated in liver tissues from NAFLD patients and steatosis mice induced by high fat diet (HFD) (63). Notably, researchers discovered that macrophages serve as the primary source of STING within the liver. Hyperactivation of STING in hepatic macrophages promotes the phosphorylation of NF-κB and JNK (c-Jun-N-terminal kinase) as well as the secretion of inflammatory factors (IL-1β, IL-6, and TNF), triggering inflammatory responses and lipid deposition in hepatocytes (63). Moreover, hyperactivation of STING in macrophages upregulates the expression of TGF-β1 and *α*-SMA, resulting in activation of hepatic stellate cells (HSC) and fibrosis (61, 63). The specific deletion of STING in macrophages attenuated hepatic fibrosis and inflammatory response. Besides, under ER stress, STING-IRF3 signaling increases the expression of pro-apoptotic molecules (such as B-cell lymphoma 2 (Bcl2)-associated X protein) as well as apoptosis promoters (such as Caspase-3), which can lead to hepatocyte apoptosis and exacerbate liver injury (64, 65). Similarly, there are reports indicating that abnormal activation of the STING-IRF3 pathway facilitates hepatocyte apoptosis and disrupts glucose and lipid metabolism, resulting in the development of NAFLD (66). Interestingly, cGAS-STING signaling was shown to enhance hepatocyte pyroptosis and hepatic inflammation in liver fibrosis through activation of NLRP3 inflammasome (67). These evidences suggest that inhibiting the hyperactivation of the cGAS-STING pathway is a potential therapeutic strategy to inhibit hepatocyte death and liver injury.

The infection of hepatitis B virus (HBV) has emerged as a worldwide public health issue. Since the cGAS-STING pathway is critical to innate immune surveillance against DNA viruses, its close association with viral hepatitis has attracted the attention from a wide range of researchers. The expression of cGAS and its effector genes was found to be down-regulated among HBV-infected hepatocytes (60). The results of a clinical study showed that STING expression was significantly reduced in peripheral blood mononuclear cells of patients with chronic hepatitis B compared with healthy controls (68). Defective expression of cGAS and STING in hepatocytes may lead to immune escape from HBV (69). Specifically, HBV polymerase disrupts K63 ubiquitination of STING and inhibits IFN-I production by interacting with STING, leading to HBV immune escape (13). Recent studies have demonstrated that the STING agonist DMXAA suppresses HBV replication and transcription by stimulating STING signaling, thereby attenuating the progression of liver fibrosis and liver injury (70).

### 3.3 cGAS/STING pathway and pancreatitis

Acute pancreatitis (AP) represents the preeminent acute inflammatory disorder within the digestive system, posing a serious health threat. The cellular mechanisms of AP mainly include abnormal reactive oxygen species accumulation, mitochondrial dysfunction, and damaged autophagy (71). As is well known, mitochondrial damage usually destroys the integrity of mitochondrial membrane, resulting in the release of mitochondrial contents into the cytoplasm, including mtDNA. Cytoplasmic escape of mtDNA significantly triggers inflammatory cascade response in AP by activating cGAS-STING pathway (72). In AP mice, STING senses DNA from dying acinar cells and stimulates pro-inflammatory signaling pathways (73). The severity of AP in STING knockout mice was significantly inferior to control mice, whereas STING agonists aggravated the condition of AP mice (73). Abnormal activation of STING signaling exacerbates the damage of intestinal mucosal barrier in severe acute pancreatitis (SAP) (74). In addition, the mtDNA-cGAS-STING pathway promotes macrophage pyroptosis through activation of IRF7/IRF3, which worsens lung injury in SAP (75). Negative regulation of molecules in this pathway inhibits NLRP3 inflammasome activation and macrophage pyroptosis, thus ameliorating SAP-associated lung injury. These findings demonstrate that cGAS-STING signaling serves a paramount role in the process by which DNA released from dead pancreatic acinar cells drives the inflammatory response in AP. Inhibiting the overactivation of the cGAS-STING pathway could be one of the potential strategies to mitigate AP.

Chronic pancreatitis (CP) is a persistent inflammation of the pancreatic parenchyma characterized by acinar cell death and persistent inflammation and fibrosis, ultimately leading to structural changes and dysfunction of the pancreas. Although both AP and CP involve immune cells sensing mtDNA released from dead acinar cells, there are significant differences in their responses to STING signaling pathway activation. Unlike AP, the activation of STING has a protective effect in CP. STING inhibits CP-associated pancreatic inflammation and fibrosis by reducing IL-17A generation during CP (76).

### 3.4 cGAS/STING pathway and digestive tumors

Strong evidence revealed a close association between DNA damage and cancer (77). “Cold” tumors exhibit an immunosuppressive tumor microenvironment (TME) with minimal immune infiltration, particularly lacking CD8^+^ T cells and natural killer cells, resulting in impaired tumor surveillance and resistance to immune checkpoint therapies (78). In contrast, “hot” tumors display immunologically active TME with robust lymphocyte infiltration and potent anti-tumor immunity. Emerging research has found that the STING signaling is fundamentally involved in triggering immune responses against tumors and the conversion of “cold” tumors into “hot” tumors (79). The generation of IFN-I facilitates immune cells to attack cancer cells and substantially strengthens the host’s resistance against tumor cells. The detection of aberrant dsDNA triggers the activation of cGAS-STING signaling, resulting in the generation of IFN-I with anti-tumor effects. Moreover, the cGAS-STING pathway facilitates complex intercellular communication among multiple immune components within the tumor microenvironment, particularly involving CD8^+^ T-cells, dendritic cells, and natural killer cells (80, 81). Notably, emerging evidence indicates that the cGAS-STING pathway plays a significant role in mediating cellular senescence in cancer cells. Senescence is an important stage in the cell life cycle, and when cells are damaged or stressed, they enter a senescent state and lose their ability to proliferate. Studies have indicated that activation of the cGAS-STING pathway can induce senescence in cancer cells (82). The ability of senescent cancer cells to proliferate and spread is blocked, facilitating the treatment of cancer.

There are many types of digestive system tumors, including gastric cancer, CRC, and esophageal cancer. The cGAS-STING pathway has been shown to be a promising target for the treatment of gastrointestinal tumors. CRC, one of the prevalent gastrointestinal cancers, ranks as the second major contributor to cancer-associated fatalities globally. Substantial evidence demonstrates that activation of the cGAS-STING signaling pathway suppresses CRC progression by inducing IFN-I-mediated immune surveillance (83, 84). Compared to CRC patients with lower STING expression, CRC patients with higher STING expression demonstrated enhanced intratumoral CD8^+^ T-cell infiltration and decreased frequency of lymphovascular infiltration during the initial stages of cancer (14). Moreover, they also had a longer overall survival period and recurrence-free survival. Further studies have indicated that intratumoral treatment with the STING agonist 3′3′-cGAMP effectively inhibits the growth of MC38 tumors, which enhances intratumoral infiltration and CD8^+^ T-cell activation (14).

*Helicobacter pylori*, a well-established exogenous carcinogen, represents a primary etiological factor in gastric cancer. Its DNA combines with cGAS to activate the cGAS-STING signaling pathway (85). Further studies have shown that patients with lower levels of STING protein expression in gastric tumor tissues exhibit worse TNM stage and diminished overall survival (85). In addition, activation of the cGAS-STING pathway inhibited gastric tumor cell proliferation, migration, and immune escape (86). Increasing studies have revealed that cGAS-STING signaling contributes to the maintenance of gastric homeostasis and exerts protective effects in gastric cancer (7). However, Miao and colleagues found that STING proteins were highly expressed in malignant tissues than normal tissues, and the high expression of STING implied a lower survival rate in gastric cancer patients (87). They found that STING was more abundantly expressed in tumor-associated macrophages (TAMs) than gastric cancer cells. With STING down-regulation or activation, STING levels in the ER decreased and macrophages polarized to pro-inflammatory subtypes and induced apoptosis in gastric cancer cells via the IL6R-JAK-IL24 pathway. The mechanisms leading to the different roles of STING in gastric cancer cells and TAM remain unknown. Further validation of this will be required for larger cohort studies and experimental studies.

Furthermore, previous studies have shown that high expression of STING in esophageal squamous cell carcinoma patients is strongly associated with decreased overall and disease-free survival (88). In contrast, another study found that STING agonists increased anti-tumor activity in combination with radiotherapy in a rat model with esophageal adenocarcinoma (89).

Pancreatic cancer is one of the deadliest and most aggressive malignancies. Studies have revealed that the STING agonist DMXAA, either alone or in combination with gemcitabine, induces significant tumor regression in both *in situ* and subcutaneous mouse pancreatic cancer models (90). The activation of STING promotes the recruitment of CD8^+^ T cells and remodels TAM by stimulating pro-inflammatory factors and chemokines, as well as activating dendritic cells (91, 92). Dronedarone hydrochloride, an antiarrhythmic drug, increases mitochondrial stress and causes mtDNA leakage, activates the cGAS-STING pathway and induces pyroptosis, which inhibits the development of pancreatic ductal adenocarcinoma in mice (93). Overall, the current study emphasizes the positive role of STING in the treatment of gastrointestinal tumors, highlighting its potential therapeutic value in enhancing anti-tumor immune responses.

## 4 Phytochemicals targeting cGAS-STING pathway to treat digestive diseases

TCM is a promising therapeutic approach for digestive diseases, owing to its high safety profile and multi-targeting properties. The compound prescriptions and active ingredients of TCM have demonstrated significant efficacy in managing digestive disorders (18). The active ingredients of TCM exhibit diverse pharmacological properties, including anti-inflammatory, antioxidant, antiviral, and anticancer effects (94, 95). These compounds, such as saponins, flavonoids, polysaccharides, polyphenols, and terpenoids, demonstrate therapeutic effects through immunomodulation, modulation of signaling pathways, and regulation of gut microbiota (96). Notably, emerging evidence suggests that specific herbal active compounds may alleviate digestive diseases by modulating the cGAS-STING pathway (97).

### 4.1 Herbal active ingredients for IBD

*Glycyrrhiza uralensis* Fisch (Gancao) is an ancient and widely utilized TCM herb with excellent efficacy in clearing heat and detoxifying. Licochalcone D, a flavonoid compound isolated from *Glycyrrhiza uralensis* Fisch, exhibits diverse pharmacological properties. It possesses anti-inflammatory, antioxidant, and antitumor effects (98–100). Licochalcone D has recently been identified as a potent and selective STING inhibitor (101). Licochalcone D was found to covalently modify the Cys148 residue of STING. By suppressing STING oligomerization, it blocks TBK1 recruitment and IRF3/NF-κB nuclear translocation, thereby slowing colitis progression and colitis-associated colon cancer (Table 1). Decursin is a coumarin analog mainly derived from the roots of *Angelica gigas* Nakai (Danggui). Decursin has been extensively studied in the field of digestive diseases (102–104). In experimental IBD mice, decursin inhibited the cGAS-STING signaling cascade response and improved the intestinal mucosal barrier (105). Furthermore, quercetin, a representative flavonoid, alleviates UC through multiple mechanisms, including suppression of cGAS-STING pathway activation, regulation of macrophage polarization (M1/M2 balance), and restoration of intestinal barrier integrity (106).

**Table 1:** Summary of herbal active ingredients that regulate the cGAS-STING pathway in digestive diseases.

Active ingredients	Experiment models	Effects	References
Licochalcone D	DSS-induced colitis mice, AOM/DSS-induced CAC mice, and dsDNA-stimulated THP-1 derived macrophages and RAW264.7 cells	↓STING oligomerization ↓p-STING, p-TBK1, p-IRF3, and p-NF-κB ↓IFNB1, CXCL10, and IL6	(101)
Decursin	DSS-induced IBD mice	↓cGAS, STING, p-STING, p-TBK1, and NF-ΚB p65, ↓TNF-α, IL-1β, and IL-6 ↑ZO-1, occludin, and claudin 1	(105)
Quercetin	DSS-induced UC mice, RAW264.7 cells, and BMDMs	↓cGAS, STING, p-TBK1, and p-IRF3 ↓M1 macrophage polarization, ↑M2 polarization ↑ZO1 and occludin	(106)
Oroxylin A	CCl_4_-induced liver fibrosis mice and human HSC line LX-2	↑cGAS, ↑IL-1β, IL-6, and IFN-β, ↑NCOA4, ↓FTH1 ↑SA-β-gal, ↑HSC senescence, ↓α-SMA and collagen 1	(107)
Oroxylin A	CCl_4_-induced liver fibrosis mice, human HSC line LX-2, and rat HSC line T6	↑SA-β-gal, ↑p16, p21 and HMGA1, ↑HSC senescence, ↑cGAS DNA demethylation, ↑cGAS, STING, p-TBK1, and p-IRF3, ↑IFN-β, ↓α-SMA and collagen 1	(108)
Naringenin	CCl_4_-induced liver fibrosis mice and Human LX2 and L02 cells	↓cGAS, STING, and IRF3, ↓IL-1β, IL-6, IL-18, and TNF-α, ↓α-SMA and collagen1	(110)
Ginsenoside Rd	CCl_4_-induced ALI mice	↓cGAS and STING ↓serum and liver iron ↓4-HNE level ↑GSH and GPX4 levels	(19)
Vanillin	Mice with maneb-induced liver damage	↓DNA fragmentation ↓MDA and H_2_O_2_ ↑SOD, GSH, and GPX4 ↓AST, ALT, ALP, GGT, and total bilirubin, ↑total protein	(113)
Schisandrin C	Hydrodynamic injection-mediated HBV-replicating mice, L929 cells, and PMA-primed THP-1 cells	↑the interaction between TBK1 and STING ↑p-STING and p-IRF3 ↑IFN-β, IFIT1, ISG15, and CXCL10 ↓HBeAg, HBcAg, HBsAg, and HBV DNA levels	(115)
Saikosaponin D	Cerulein-treated AR42J cells (PAC injury model)	↓mtDNA release, ↓cGAS and STING ↓NLRP3 and cleaved-caspase-1	(119)
Icariside I	Mouse colon carcinoma (CT26) and MFC cell lines, CT26 colon carcinoma bearing mice, and MFC gastric cancer bearing mice	↑mtDNA release, ↑cGAS, STING, and p-IRF3, ↑IFN-β	(121)
Andrographolide	Irinotecan-induced colitis in tumor-bearing mice	↓dsDNA, ↓p-TBK-1 and p-IRF3 ↓IFN-β, CXCL10, and CCL5 ↓Il-1β, TNF-α, and IL-18	(97)
Naringin	Ischemia/reperfusion-injured rat and hypoxia-reoxygenation-injured IEC-6 cells	↓IL-6, IL-1β, TNF-α, and IFN-β, ↑SOD and GSH, ↓MDA ↓cGAS, STING, p-TBK1, p-IRF3, and NF-κB	(128)

ALI, acute liver injury; ALP, alkaline phosphatase; ALT, alanine aminotransferase; AST, aspartate aminotransferase; BMDMs, bone marrow-derived macrophages; CAC, colitis-associated colon cancer; CCl_4_, carbon tetrachloride; CRC, colorectal cancer; DSS, dextran sodium sulfate; GGT, gamma-glutamyl transpeptidase; GPX4, glutathione peroxidase 4; GSH, glutathione; H_2_O_2_, hydrogen peroxide; HFD, high fat diet; HSC, hepatic stellate cell; IBD, inflammatory bowel diseases; IFIT1, IFN-induced proteins with tetratricopeptide repeat 1; IL-6, interleukin-6; IRF3, interferon regulatory factor 3; ISG15, IFN-stimulated gene 15; MDA, malondialdehyde; MFC, forestomach carcinoma; NAFLD, non-alcoholic fatty liver disease; PACs, pancreatic acinar cells; SOD, superoxide dismutase; TBK1, TANK-binding kinase 1; UC, ulcerative colitis.

### 4.2 Herbal active ingredients for liver diseases

Oroxylin A is an active flavonoid extracted from *Scutellaria baicalensis* Georgi (HuangQin). Interestingly, oroxylin A activates ferritinophagy through upregulation of the cGAS-STING pathway and accelerates HSCs senescence, thus suppressing liver fibrosis (107). Another study found that oroxylin A suppressed methylation of the cGAS gene through inhibiting the formation of methionine metabolites, promoting HSC senescence (108). Besides, naringenin, a flavonoid with anti-inflammatory properties, has numerous health advantages. Naringenin was shown to attenuate hepatic fibrosis in carbon tetrachloride (CCl_4_)-induced rats by attenuating endoplasmic reticulum stress and suppressing excessive autophagy (109). In CCl_4_-treated hepatic fibrosis mice, naringenin significantly attenuated liver injury, collagen deposition, and cGAS expression (110). Molecular docking identified cGAS as a direct binding target of naringenin, and *in vitro* studies confirmed its ability to suppress cGAS-STING-driven inflammation in activated HSCs.

*Panax ginseng* C. A. Meyer. (Renshen) is a famous Chinese herb that occupies a highly significant position in TCM and is widely used in Asian countries. Ginsenoside Rd, an essential active ingredient of *Panax ginseng* C. A. Meyer, belongs to tetracyclic triterpenoid derivatives. Ginsenoside Rd shows various biological activities, including anti-inflammatory, antitumor, neuroprotective, hepatoprotective, cardiovascular protective, and immunomodulatory properties, making it a promising candidate for the treatment of diverse diseases (94, 95, 111). Notably, ginsenoside Rd was shown to attenuate acute hepatic damage by inhibiting ferroptosis mediated by cGAS-STING pathway (19). Ginsenoside Rd improved serum and liver iron levels, as well as lipid peroxidation, in mice with acute liver damage. Additionally, ginsenoside Rd significantly elevated both glutathione and glutathione peroxidase 4 levels. Notably, it reduced the expression levels of cGAS and STING. Vanillin is a natural phenolic compound derived from vanillin beans. In rats with thioacetamide-induced liver fibrosis, vanillin demonstrated promising therapeutic effects by attenuating hepatic fibrosis and promoting liver regeneration (112). Vanillin promoted the recovery of liver function, significantly reducing ALT and AST levels while increasing serum albumin and total protein levels. Its hepatoprotective activity is mediated by antioxidant effects (reduced MDA and increased GSH) and anti-inflammatory effects (suppressed IL-6 and TNF-α). Furthermore, vanillin significantly attenuated DNA fragmentation and reduced oxidative stress in mice with maneb-induced liver injury (113).

*Schisandra chinensis* (Wuweizi) is a widely used traditional Chinese herb with effects of tonifying the kidneys and calming the heart, tonifying qi and engendering fluid. “Tonifying qi” is a TCM concept denoting enhancement of bioenergy metabolism and immune regulation. Pharmacological studies have indicated that *Schisandra chinensis* modulates host immunity and exhibits anticancer, antiviral, and hepatoprotective functions (114). Schisandrin C, one of the active components of *Schisandra chinensis*, promotes the activation of the cGAS-STING signaling pathway by facilitating the interaction between TBK1 and STING, thereby inhibiting HBV replication (115). Another study indicated that the combination of luteolin and schisandrin C had synergistic anti-HBV effects in mice (116). The synergy results from luteolin’s direct antiviral effect (inhibiting virus reproduction) and schisandrin C’s immunological activation (augmenting innate antiviral defense). Luteolin showed significant anti-HBV action by reducing HBV replication through the downregulation of hepatocyte nuclear factor 4α via the ERK pathway. Schisandrin C enhanced the activation of cGAS-STING pathway and the generation of IFN-β in macrophages to suppress HBV replication in HepG2.2.15 cells.

### 4.3 Herbal active ingredients for pancreatitis

Saikosaponin D is a triterpenoid saponin compound derived from *Bupleuri radix* (Chaihu), exhibiting anti-inflammatory, anticancer, and antiviral properties (117). Saikosaponin D has exhibited considerable protective effects against pancreatic injury in CP by attenuating acinar cell apoptosis and inflammation in both *in vivo* and *in vitro* models (118). Mechanistically, saikosaponin D inhibited the activation of MAPK signaling pathways in pancreatic acinar cells. A recent study found that saikosaponin D attenuated cerulein-induced pancreatic acinar cell damage by inhibiting NLRP3/caspase-1-mediated pyroptosis (119). Notably, it mitigated mitochondrial damage, reduced oxidative stress, and suppressed mtDNA release, thereby inhibiting cGAS-STING pathway activation.

### 4.4 Herbal active ingredients for digestive tumors

Icariside I is a flavonoid extracted from the traditional Chinese herb *Epimedii folium* (Yinyanghuo). Icariside shows unique potential in the field of tumor immunotherapy (120). It represents a promising candidate to overcome the limitations of immune checkpoint inhibitors by remodeling the immunosuppressive TME. A recent study found that icariside I enhanced the efficacy of immunotherapy for gastrointestinal cancers (121). Mechanistically, icariside I binds to receptor potential vanilloid 4 (TRPV4), triggering intracellular Ca^2+^ influx and mitochondrial oxidative stress, which promotes the release of oxidized mtDNA into the cytosol. These mtDNA fragments are subsequently engulfed by immune cells within the TME, activating the cGAS-STING pathway and amplifying anti-tumor immunity.

### 4.5 Herbal active ingredients for other digestive diseases

Andrographolide is a dicyclic diterpene lactone derived from *Andrographis paniculata* (Burm.f.) Nees (Chuanxinlian), which is widely used worldwide. Andrographolide has numerous pharmacological efficacies, particularly notable for its anti-inflammatory, antioxidant, antiviral, antitumor, and immunoregulatory activities (97, 122, 123). Andrographolide alleviates UC by mitigating oxidative stress, suppressing inflammation, and inhibiting Th1/Th17 responses (124, 125). Recently, Wang et al. (97) found that andrographolide promotes homologous recombination repair and inhibits dsDNA-GAS-STING signaling, thereby ameliorating gastrointestinal mucositis induced by the chemotherapeutic drug irinotecan.

Naringin is a natural flavanone glycoside, one of the primary active components in citrus herbs. Naringin exhibits diverse therapeutic properties, involving anti-inflammatory, anti-apoptotic, and hepatoprotective activities (126, 127). *In vivo* and *in vitro* experiments demonstrated that naringin alleviated oxidative damage in intestinal ischemia-reperfusion models by enhancing superoxide dismutase and glutathione activity while suppressing malondialdehyde production (128). In addition, naringin inhibited inflammation and apoptosis. Importantly, naringin inhibited the activation of cGAS-STING signaling. cGAS silencing down-regulated cGAS-STING pathway-related proteins and partially attenuated the protective effects of naringin, indicating that naringin’s efficacy depends on this pathway.

## 5 Conclusion and perspective

The cGAS-STING pathway, a crucial element of innate immunity, responds to multiple intrinsic and extrinsic stimuli, triggering and amplifying inflammatory processes. The activation of the cGAS-STING signaling pathway, along with a number of downstream pro-inflammatory mediators, constitutes a fundamental mechanism underlying inflammatory cascades while contributing significantly to the pathogenesis of various gastrointestinal disorders. The cGAS-STING signaling has emerged as a promising molecular target for diverse gastrointestinal disorders. This review summarized herbal active ingredients used to treat digestive disorders by affecting the cGAS-STING pathway, including licochalcone D, ginsenoside Rd, schisandrin C, and quercetin. They primarily used to the management of IBD, liver fibrosis, pancreatitis, CRC, gastric cancer, and other diseases. The therapeutic effect is achieved by up-regulating or down-regulating the expression of cGAS-STING in relevant tissues and organs.

However, research investigating the regulatory mechanisms of the cGAS-STING signaling in digestive diseases remains at an early phase. Large-scale cohort and experimental studies are essential to validate these preliminary observations and establish conclusive evidence. Secondly, the cGAS-STING pathway may exert dual effects in digestive diseases. The dual roles of cGAS-STING in digestive diseases likely result from its context-dependent regulation, where tissue microenvironment, disease duration, and cellular composition collectively determine functional outcomes. The specific mechanisms leading to these differences deserve further investigation. Deciphering these context-dependent effects allows for precise therapeutic interventions ailored to specific disease stages or cell types. Moreover, the role of the cGAS-STING pathway in digestive autoimmune diseases, such as autoimmune gastritis and autoimmune hepatitis, remains largely unexplored, with significant research gaps persisting in this field. Future studies should investigate the role of the cGAS-STING pathway in these diseases. At present, the regulation of the cGAS-STING signaling by plant-derived compounds remains at the preclinical research phase. Current research on active compounds has predominantly investigated their effects on the cGAS-STING pathway, while largely neglecting systematic research of their structure-activity relationships. Additionally, many herbal active components have low rates of absorption, solubility, stability and overall bioavailability, which largely limits their clinical applications. Nanoparticle-based drug delivery systems represent a promising strategy to overcome these limitations.
